# Comparison of Adhesion of Immortalized Human Iris-Derived Cells and Fibronectin on Phakic Intraocular Lenses Made of Different Polymer Base Materials

**DOI:** 10.3390/medicina61081384

**Published:** 2025-07-30

**Authors:** Kei Ichikawa, Yoshiki Tanaka, Rie Horai, Yu Kato, Kazuo Ichikawa, Naoki Yamamoto

**Affiliations:** 1 Chukyo Eye Clinic, Nagoya 456-0032, Aichi, Japan; kei@chukyogroup.jp (K.I.); ytanaka@chukyomedical.co.jp (Y.T.); horai@chukyomedical.co.jp (R.H.); 2 General Aoyama Hospital, Toyokawa 441-0103, Aichi, Japan; 3 Center for Society-Academia Collaboration, Research Promotion Headquarters, Fujita Health University, Toyoake 470-1192, Aichi, Japan; yu.toki@fujita-hu.ac.jp (Y.K.); naokiy@fujita-hu.ac.jp (N.Y.); 4 International Center for Cell and Gene Therapy, Research Promotion Headquarters, Fujita Health University, Toyoake 470-1192, Aichi, Japan

**Keywords:** implantable contact lens (ICL), phakic implantable contact lens (Phakic-ICL), collamer, implantable phakic contact lens (IPCL), LENTIS comfort, fibronectin (FN)

## Abstract

*Background and Objectives:* Posterior chamber phakic implantable contact lenses (Phakic-ICL) are widely used for refractive correction due to their efficacy and safety, including minimal corneal endothelial cell loss. The Collamer-based EVO+ Visian implantable contact lens (ICL), manufactured from Collamer, which is a blend of collagen and hydroxyethyl methacrylate (HEMA), has demonstrated excellent long-term biocompatibility and optical clarity. Recently, hydrophilic acrylic Phakic-ICLs, such as the Implantable Phakic Contact Lens (IPCL), have been introduced. This study investigated the material differences among Phakic-ICLs and their interaction with fibronectin (FN), which has been reported to adhere to intraocular lens (IOL) surfaces following implantation. The aim was to compare Collamer, IPCL, and LENTIS lenses (used as control) in terms of FN distribution and cell adhesion using a small number of explanted Phakic-ICLs. *Materials and Methods:* Three lens types were analyzed: a Collamer Phakic-ICL (EVO+ Visian ICL), a hydrophilic acrylic IPCL, and a hydrophilic acrylic phakic-IOL (LENTIS). FN distribution and cell adhesion were evaluated across different regions of each lens. An in vitro FN-coating experiment was conducted to assess its effect on cell adhesion. *Results*: All lenses demonstrated minimal FN deposition and cellular adhesion in the central optical zone. A thin FN film was observed on the haptics of Collamer lenses, while FN adhesion was weaker or absent on IPCL and LENTIS surfaces. Following FN coating, Collamer lenses supported more uniform FN film formation; however, this did not significantly enhance cell adhesion. *Conclusions*: Collamer, which contains collagen, promotes FN film formation. Although FN film formation was enhanced, the low cell-adhesive properties of HEMA resulted in minimal cell adhesion even with FN presence. This characteristic may contribute to the long-term transparency and biocompatibility observed clinically. In contrast, hydrophilic acrylic materials used in IPCL and LENTIS demonstrated limited FN interaction. These material differences may influence extracellular matrix protein deposition and biocompatibility in clinical settings, warranting further investigation.

## 1. Introduction

A phakic implantable contact lens (Phakic-ICL) is a lens inserted into the eye to correct refractive errors while preserving the natural crystalline lens. Phakic-ICL implantation is a reversible surgical procedure that offers a treatment option for patients with high myopia or corneal abnormalities, such as irregular shape or insufficient thickness. If necessary, the Phakic-ICL can be removed or replaced. It is positioned in the posterior chamber (the space between the iris and crystalline lens) on the posterior surface of the iris. Phakic-ICL implantation was developed in the 1980s, predating the first report of LASIK in 1990 [1,2].

The Implantable Phakic Contact Lens (IPCL) consists of a hybrid semi-hydrophilic acrylic material designed to maintain visual function over extended periods. This material contains high water content and resists the adhesion of foreign substances, such as proteins, to the lens surface [3]. The IPCL has a large optical zone (6.6 mm, diameter) and a trapezoidal aperture rather than a cylindrical one, which helps reduce halos and glare. Seven holes in the optic zone promote aqueous humor circulation, reducing the risk of cataracts and glaucoma. However, since the IPCL material has no affinity for fibronectin (FN), proteins from the aqueous humor may adhere to the lens surface, potentially causing the immune system to recognize it as a foreign body and trigger an immune response. This phenomenon has been observed with other lenses manufactured from hybrid semi-hydrophilic acrylic materials [4].

Two representative Phakic-ICLs are the Implantable Collamer^®^ Lens (Collamer), manufactured by STAAR Surgical, and the IPCL, manufactured by Care Group Sight Solution [5]. The ICL is composed of Collamer, a hydrophilic material made of a copolymer grafted with collagen. This material promotes FN adhesion and enhances hydrophilicity. The lens includes a central port that facilitates gas and nutrient exchange, reducing the risk of cataracts and glaucoma [6]. Collamer consists of a copolymer of hydroxyethyl methacrylate (HEMA) and a UV-absorbing methacrylic monomer (benzophenone), with a small amount of collagen. The material contains 36% water and has <1 ppm of residual monomers in its final hydrated state [7]. Its hydration is uniform throughout the lens. Due to the collagen component, Collamer exhibits affinity for FN, but the limited amount of collagen does not promote broader protein or cell adhesion. Instead, it supports the formation of a monolayer of FN on the lens surface, which inhibits the binding of other aqueous proteins. Because this FN layer is derived from the host, the immune system does not recognize the lens as a foreign body [8,9,10,11]. Due to the presence of collagen, studies have reported that FN binds relatively significantly to various cell-extracellular matrix (ECM) interfaces, making it difficult for other ECM proteins to adhere [11].

ECM interactions are complex processes. The ECM not only provides structural support for cells and tissues but also transmits biochemical signals through membrane-spanning receptors that regulate cell proliferation, differentiation, migration, and adhesion. Increased hydrophilicity of a material reduces interfacial energy with water while increasing surface free energy, both of which contribute to reduced cell adhesion. Maximum cell adhesion occurs at a water contact angle of 60° to 70°. Both high hydrophilicity and excessive hydrophobicity can suppress cell adhesion [12]. FN is one of the ECM proteins present in the eye.

Among Phakic-ICLs, posterior chamber lenses are selected for their efficacy and safety, including minimal corneal endothelial cell loss. Collamer lenses have dominated this market sector due to their established safety profile. However, several companies have recently introduced hydrophilic acrylic ICLs. In this study, we investigated the safety characteristics of these newer lenses, focusing on differences in material composition and design. We compared the two most widely used Phakic-ICL, Collamer ICL, and IPCL, regarding their interaction with FN, which forms part of the cellular matrix, including their cellular adhesion properties.

## 2. Materials and Methods

### 2.1. Materials

Two types of Phakic-ICL were used in the experiments: Collamer lens (EVO+ VISIAN^®^ Implantable Collamer^®^ Lens VICM5_12.6, STAAR Surgical Co., Lake Forest, CA, USA) with a length of 12.6 mm, an optic diameter of 5.0–6.1 mm, and a power of –7.50 diopters; and IPCL lens (Implantable Phakic Contact Lens V2.0, Care Group Sight Solution LLP, Gujarat, India) with a length of 12.5 mm, an optic diameter of 6.60 mm, and a power of –7.50 diopters. Additionally, one type of Phakic-ICL was included in this study: LENTIS (LENTIS^®^ Comfort Acrylic Foldable Intraocular Lens LS-313 MF15, Santen Pharmaceutical Co., Ltd., Osaka, Japan), with a length of 11.0 mm, an optic diameter of 6.0 mm, and a power of +10.0 diopters. The LENTIS lens, manufactured from hydrophilic acrylic material, served as a control for comparison with the Collamer and IPCL lenses in this study (Supplementary Table S1).

### 2.2. Explanted Lenses

We analyzed the surface characteristics of explanted lenses from three cases: two involving Collamer lenses and one involving an IPCL (Table 1).

- Case 1: A patient with a Collamer lens implanted 171 months previously.
- Case 2: A patient with a Collamer lens implanted 178 months previously.
- Case 3: A patient with an IPCL implanted 16 months previously.

This retrospective study was approved by the Medical Research Ethics Committee (Chukyo Eye Clinic Ethics Committee, No. 20250519095) and adhered to the tenets of the Declaration of Helsinki. In this study, Phakic-ICLs that were removed and would otherwise be discarded were used as research materials; therefore, there was no invasion or intervention. We obtained consent from patients to use the removed Phakic-ICLs and medical information under the condition that all data would be anonymized to prevent patient identification. Furthermore, following ethics committee review and given that only anonymized patient and medical information were used retrospectively, we disclosed the research content of this study using an opt-out method.

### 2.3. Immunohistochemical Staining of FN Using Explanted Phakic-ICL

After explantation, each lens was immediately placed in fresh BSS Plus solution (Alcon Japan Ltd., Tokyo, Japan), washed, and temporarily stored. The lenses were then fixed in 4% paraformaldehyde (FUJIFILM Wako Pure Chemical Corp., Osaka, Japan) at room temperature for 15 min, followed by a 15 min wash in phosphate-buffered saline (PBS, FUJIFILM Wako Pure Chemical Corp.) at room temperature. A protein blocking solution (Cat. No. X0909, Agilent Technologies, Inc., Santa Clara, CA, USA) was applied at room temperature for 10 min. Excess blocking solution was aspirated, and rabbit polyclonal anti-fibronectin antibody (Cat. No. ab2413, 1:50 dilution, Abcam plc., Cambridge, UK) was added and incubated overnight at 4 °C. After washing with PBS, the fluorescent secondary antibody, Donkey anti-Rabbit IgG (H + L), Highly Cross-Adsorbed, Alexa Fluor™ 488 (Cat. No. A-21206, 1:500 dilution, Thermo Fisher Scientific Inc., Waltham, MA, USA), was applied and incubated at 37 °C for 2 h. The samples were then washed with PBS, observed, and photographed using a fluorescence inverted microscope (Olympus Power IX71, Olympus Corp., Tokyo, Japan) and a digital camera system (Olympus DP-51, Olympus Corp.).

### 2.4. Experimental FN Fluorescence Detection

Five micrograms (5 µg) of preliminarily green fluorescently labeled FN (HiLyte Fluor™ 488-labeled, Cytoskeleton, Inc., Denver, CO, USA) was dissolved in 1 mL of BSS, then added to a Falcon^®^ 24-well cell culture plate (Corning Incorporated, Corning, NY, USA), with one well used for each lens. Three types of lenses were tested: lenses precoated with FN (three lenses per type) and lenses in BSS only (one lens per type). The lenses were incubated at 37 °C for 36 h. After incubation, the lenses were thrice washed with BSS before observation. FN fluorescence intensity on the lenses was measured using the real-time imaging microplate reader Spark^®^ Cyto (Tecan Group Ltd., Männedorf, Zürich, Switzerland), and further analyzed using an inverted fluorescence microscope (Olympus Power IX71, Olympus Corp.) and digital camera system (Olympus DP-51, Olympus Corp.). Each lens was tested three times in a single experiment, and the same experiment was repeated twice.

### 2.5. Cell Adhesion Experiments

The lenses used were Collamer, IPCL, and LENTIS lenses, which were precoated under the same conditions as the FN adhesion experiments (5 µg/mL in BSS) or placed in BSS only. A total of 18 lenses of each type were used.

Immortalized human iris epithelial cells (iHIE-NY2) were used in cell adhesion experiments because phakic ICLs may come into contact with iris epithelial cells. Briefly, a portion of iris tissue removed during partial iridectomy from patients with angle-closure glaucoma was cultured. The culture conditions, gene transfer method, and vector information were as previously reported [13,14]. Cells were seeded by adding 100 µL of cell suspension at a concentration of 1 × 10^5^ cells/mL per lens and allowing them to stand for 2 h before adding 1 mL of culture medium.

Cell growth was observed and recorded using an inverted microscope (Olympus Power IX71, Olympus Corporation, Tokyo, Japan) and a digital camera system (Olympus DP-51, Olympus Corporation). Cell proliferation was measured using Cell Counting Kit-8 (Dojindo Laboratories Co., Ltd., Kumamoto, Japan), containing 2-(2-methoxy-4-nitrophenyl)-3-(4-nitrophenyl)-5-(2,4-disulfophenyl)-2H-tetrazolium monosodium salt (WST-8). Cultures were performed in triplicate for each sample, and cell adhesion and proliferation were evaluated after 1, 5, and 10 days of culture.

Cell counting Kit-8 reagent, diluted 1:10 in culture medium, was added to each well and allowed to react for 3 h at 37.0 °C in a 5% CO_2_ incubator. The supernatant from the culture medium was then transferred to a 96-well, clear, TC-treated multiple-well plate (P/N: 3599, Corning Inc.), and the absorbance at 450 nm was measured using a microplate reader (Multiskan FC, Thermo Fisher Scientific Inc., Waltham, MA, USA). Five lenses were used in each experiment, with one lens serving as a negative control for each lens type. The same experiment was repeated three times.

### 2.6. Statistical Analysis

Data are presented as mean ± standard deviation (SD) and were analyzed using Kruskal–Wallis one-way ANOVA, followed by Scheffé’s post hoc test for comparisons among three or more independent groups, using SPSS Statistics 24 (IBM Corporation, New York, NY, USA).

## 3. Results

The following results describe the FN adhesion status in explanted Collamer, IPCL, and LENTIS lenses, FN adhesion in experimental lenses, and cell adhesion to lenses with and without FN coating.

### 3.1. FN and Cell Adhesion Status in Explanted Lenses

**Figure 1:**
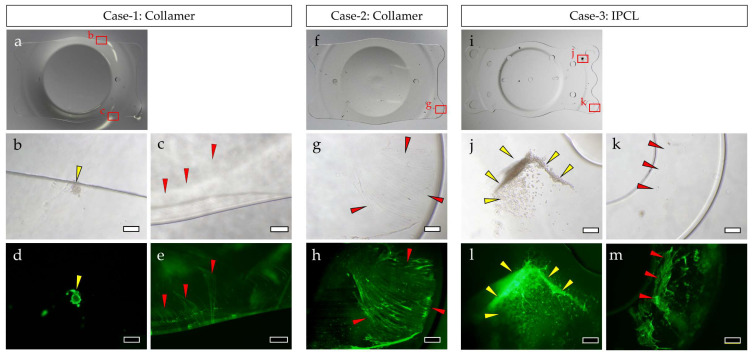
Surface analysis of explanted ICLs: Collamer explanted in Case 1 (**a**–**e**), Collamer explanted in Case 2 (**f**,**g**), and IPCL explanted in Case 3 (**i**–**m**). Macrograph (**a**,**f**,**i**), phase contrast microscope photographs (**b**,**c**,**g**,**j**,**k**), and fluorescent microscope photographs of FN stained with antibody (**d**,**e**,**h**,**l**,**m**). Images show the same locations (**b** and **d**, **c** and **e**, **g** and **h**, **j** and **l**, **k** and **m**). Yellow arrows: cellular components. Red arrows: FN. Bar = 200 µm (**b**,**d**,**g**,**h**,**j**,**k**,**l**,**m**) or bar = 500 µm (**c**,**e**).

In both Collamer and IPCL explants, the central optical zone area was almost devoid of FN and cellular components. In contrast, FN and cellular components were observed adhering to the four haptics in Collamer and to portions of the six haptics in IPCL, as well as at the peripheral areas where the lens contacts the iris. In Case 1, slight cellular components adhered to the iris-contact area, and a thin FN film was observed in that region. In Case 2, FN adhered as a thin film, particularly on the haptics. In Case 3, cell aggregates were observed on some haptics, and FN was observed adhering to the haptics, though not uniformly distributed (Figure 1).

### 3.2. Evaluation of FN Adhesion

**Figure 2:**
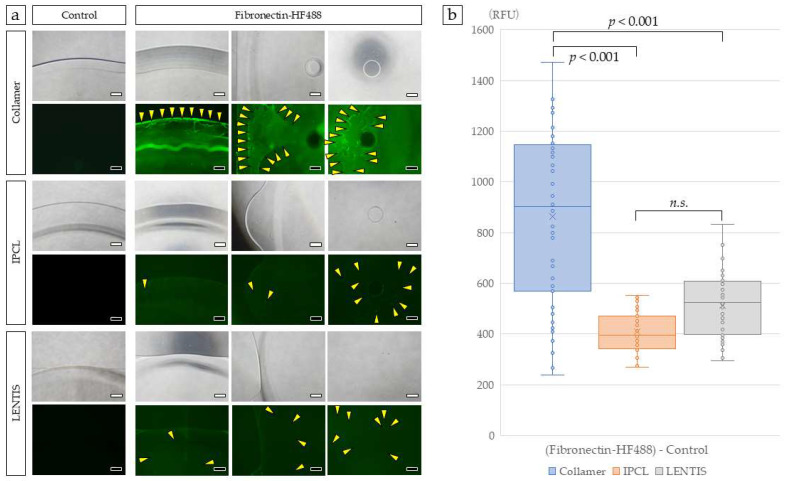
Surface analysis of ICLs incubated with FN. Collamer demonstrated film-like fluorescence at the optic edge, mid-optic region, and optic center. Slight green fluorescence was observed in IPCL and LENTIS compared to controls. Yellow arrowheads indicate FN detected as discrete points (**a**). Each lens was photographed with a 40× objective lens. Fluorescence intensity was quantified using a real-time imaging microplate reader (**b**). Statistical analysis was performed using Kruskal–Wallis one-way ANOVA. Bar = 500 µm. The n.s. was not significant.

After incubation with fluorescently labeled FN solution, FN adhered to the optical edge and center of the Collamer lens optic, as observed using fluorescence microscopy. The Collamer lens demonstrated film-like FN distribution across these regions. In contrast, IPCL and control LENTIS lenses showed increased fluorescence compared to uncoated lenses, but the FN distribution was not uniform or film-like in pattern (Figure 2a). When fluorescently labeled FN was quantified using a real-time imaging microplate reader, Collamer demonstrated the highest fluorescence intensity, significantly greater than that observed in IPCL and LENTIS lenses (Figure 2b).

### 3.3. Effect of FN Coating on Cell Adhesion and Proliferation

iHIE-NY2 cells were seeded onto FN-coated and uncoated Collamer, IPCL, and LENTIS lenses and cultured at 37 °C in a 5% CO_2_ incubator for 1, 5, and 10 days. Positive controls were established by culturing the same number of cells in standard cell culture wells. Cell adhesion was generally enhanced by FN coating across all three lens types.

**Figure 3:**
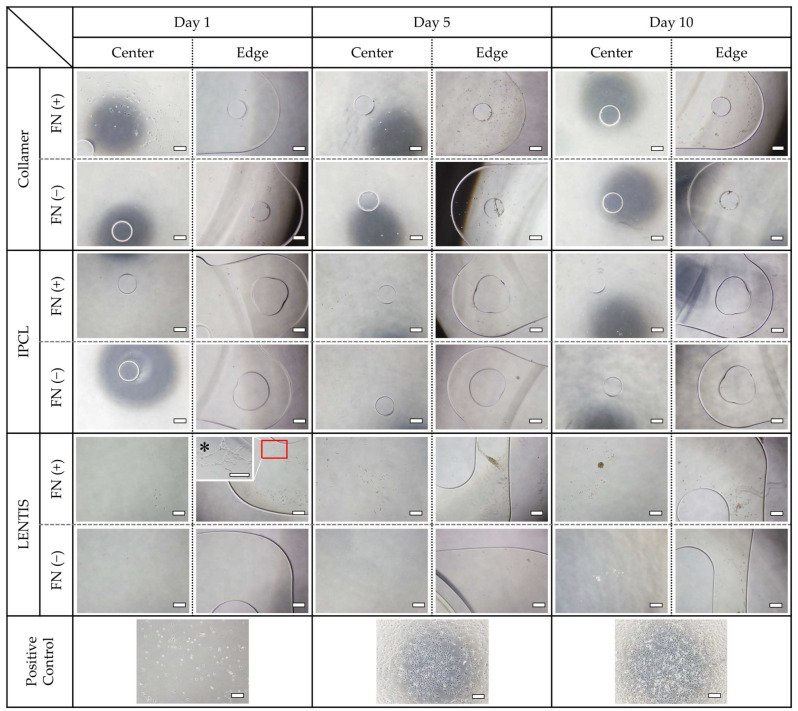
Cell adhesion to each lens with and without FN coating. Phase contrast images of the optic center and haptics on days 1, 5, and 10 of culture. Positive controls were cultured in standard cell culture wells with the same number of seeded cells. All images were taken with a 40× objective lens. * Magnification of the haptics of FN-coated LENTIS was taken with a 100× objective lens. Bar = 500 µm.

On Collamer lenses, cells adhered to the optic center on day 1, but the number of adherent cells decreased by day 10. In contrast, the haptics demonstrated gradual cell proliferation, with marked adhesion observed within the haptic holes. IPCL lenses exhibited fewer adherent cells in both the optic center and haptics compared to Collamer. Minimal cell adhesion was observed within the haptic holes of IPCL. LENTIS lenses also demonstrated minimal adhesion to the optic surface; however, sheet-like clusters of adherent cells were observed in some haptic areas. The number of adherent cells on both IPCL and LENTIS was significantly lower than in positive control wells (Figure 3). The haptic holes of Collamer appeared smaller and had rougher inner surfaces compared to IPCL, which had larger, more rounded holes.

**Figure 4:**
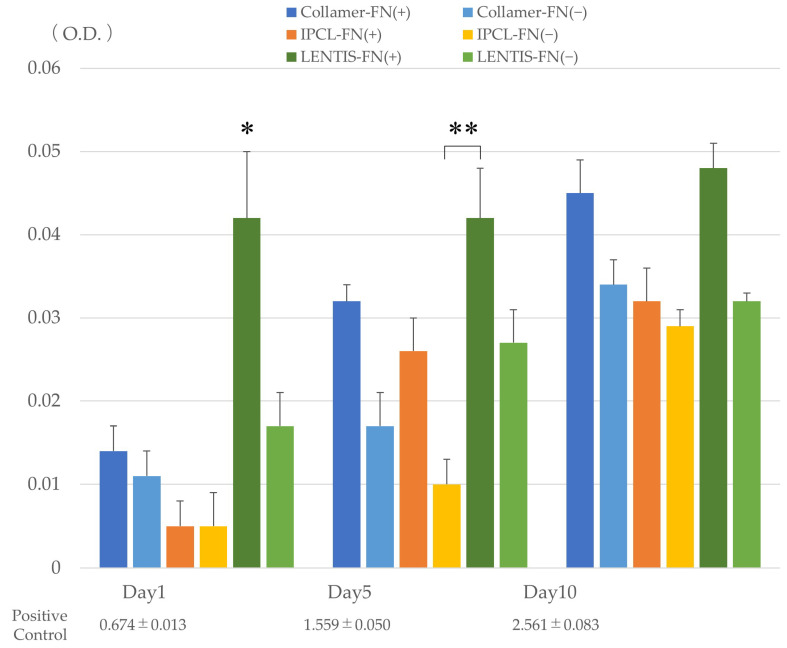
Cellular activity on each lens with or without FN coating measured using WST-8 on days 1, 5, and 10 of incubation. * Significant difference (*p* < 0.05) between FN-coated LENTIS lenses and other lenses on day 1. ** Significantly lower cell activity (*p* < 0.05) in uncoated IPCL lenses compared to FN-coated LENTIS lenses on day 5. The positive control represents cell activity in standard culture wells seeded with the same number of cells. O.D. indicates optical density (absorbance). Statistical analysis was performed using Kruskal–Wallis one-way ANOVA.

Cell activity, as measured by WST-8, demonstrated a slight overall increase over time across all lens types. On day 1, cell activity was significantly lower in uncoated Collamer, IPCL, and LENTIS lenses compared to the FN-coated LENTIS lens (*p* < 0.05). On day 5, only the uncoated IPCL lens showed significantly lower activity than FN-coated LENTIS lenses (*p* < 0.05). By day 10, no significant differences in cell activity were observed between lens types or coating conditions (Figure 4). Using FN-coated LENTIS as a reference, all lens types demonstrated increased cellular activity over time. Across all lens types, FN-coated lenses consistently exhibited higher activity than their uncoated counterparts (Table 2). Statistically significant differences were observed in cell adhesion and cell activity based on the presence or absence of FN coating. However, while the FN-coated LENTIS control demonstrated the highest cell adhesion among all experimental groups, the increases in cell adhesion and cell activity across all lens types were only slight, and no visible lens opacification due to cell adhesion was observed.

## 4. Discussion

Phakic-ICLs provide superior visual quality, faster recovery, greater refractive accuracy and stability, preservation of accommodation, and reversibility compared to corneal refractive surgeries such as LASIK [9,15,16].

Collamer is a copolymer comprising HEMA and grafted collagen. It has a soft texture due to the unique mechanical properties of its polymer meshwork (Poisson’s ratio: 0.4999; elongation at break: 1000%). The presence of collagen imparts a negative surface charge, repelling negatively charged proteins and reducing biofilm formation. Collamer demonstrates excellent biocompatibility and maintains long-term intraocular stability. ICLs manufactured from Collamer received CE marking in Europe in 2003, FDA approval in the U.S. in 2005, and the Ministry of Health, Labour and Welfare approval in Japan in 2010. ICL surgery is now performed in over 70 countries and is recognized alongside LASIK as a standard refractive correction procedure. The Collamer ICL has undergone several design iterations to achieve its current optimized form [6,17,18].

In the early 1990s, Fyodorov of the Moscow Eye Institute introduced the posterior chamber Phakic-ICL. His silicone model, a predecessor of the PRL (Phakic Refractive Lens, Carl Zeiss Meditec), was discontinued due to cataract formation and zonular damage, as the material was too rigid (Poisson’s ratio: 0.47) for delicate intraocular tissues. This experience led to the development of the much softer Collamer material at the same institute. Incorporating collagen into Collamer improved its biocompatibility by enabling FN monolayer formation on the lens surface, effectively shielding it from immune recognition [19]. Furthermore, the FN monolayer formed on Collamer may contribute to the rotational stability of the intraocular lens, although further investigation is needed regarding this hypothesis.

Our focus on FN in this study was based on previous research in the ophthalmic field demonstrating that ECM components continuously affect cellular behavior when corneal epithelial cells are cultured on plates coated with laminin, FN, and type IV collagen [20]. Furthermore, the major differences between Collamer and IPCL are their base materials, HEMA versus acrylic, and the presence or absence of collagen. It has been reported that covalently bonding collagen to HEMA-based Phakic-ICLs of similar shape to Collamer results in FN inhibiting the non-specific adsorption of other proteins [10].

The IPCL comprises a hybrid hydrophilic acrylic material with high water content, designed to minimize protein adhesion and maintain long-term visual function. It has a large (6.6 mm) optical zone and a trapezoidal aperture to reduce halos and glare. Seven peripheral holes improve aqueous humor circulation, decreasing the risks of cataract and glaucoma formation [3]. However, compared to Collamer, our study results demonstrated that IPCL materials lack affinity for FN. Consequently, proteins from aqueous humor can adhere to the lens surface, potentially triggering immune recognition and inflammatory responses [8,9,10,11].

The ECM provides a structural scaffold for cell adhesion. FN binds to collagen in the ECM, while integrins on cell membranes bind to FN, facilitating cell attachment. However, highly hydrophilic surfaces tend to have lower interfacial energy and higher surface free energy, which weakens cell adhesion. Therefore, hydrophilic lenses such as IPCL may hinder cell attachment [12].

This study focused on the effect of FN in cell adhesion. While FN and bovine serum albumin enhance cell adhesion on various materials, HEMA alone demonstrates minimal adhesion under most conditions [8]. However, in Collamer lenses, FN adheres to the grafted porcine collagen, forming a protective membrane-like layer. In contrast, FN adhered only in discrete patches to IPCL and LENTIS lenses. Since FN adhesion increases on more hydrophilic surfaces (those with lower contact angles), our findings suggest that Collamer‘s superior FN adhesion reflects its more hydrophilic properties among the lenses studied [21].

Cell adhesion was significantly lower on all lenses compared to positive controls. Within individual lenses, the haptic regions tended to support greater cell adhesion than the optic regions, a trend that was enhanced by FN coating. Additionally, cells adhered to the inner surfaces of the haptic holes in Collamer lenses. These holes are smaller than those in IPCL, and differences in inner surface texture and overall geometry may influence adhesion patterns. Although LENTIS is an IOL rather than a Phakic-ICL, it was included as a material control because it is manufactured from hydrophilic acrylic material similar to IPCL. The haptics of Phakic-ICLs are inserted into the narrow space between the iris and crystalline lens. Since the iris is constantly mobile due to factors such as light exposure, inflammation caused by lens material poses a risk of adhesion to the iris tissue. The explanted Collamer lenses demonstrated membrane-like FN attachment to the haptics without iris adhesion, allowing successful extraction. These results suggest that although FN attachment occurred, cellular adhesion remained minimal, maintaining biocompatibility and allowing sustained transparency over the long term. Furthermore, in 342 eyes examined 3 yrs after Collamer implantation, laser flare photometry and cellular response measurements, which quantify anterior chamber inflammation, showed zero flare values and cellular responses, indicating excellent biocompatibility [11]. Based on our study results, Collamer demonstrated high FN adhesion and attachment properties, but we attribute the lack of increased cell adhesion to the inherent properties of both the collagen component and the HEMA base material. We hypothesize that improved FN biocompatibility may lead to reduced foreign body reactions over the long term, as fewer deposits accumulate on the lens surface, thereby maintaining Collamer transparency. The lack of increased cell adhesion is attributed to the inherent properties of HEMA materials, which are naturally less conducive to cell adhesion.

Surface modification of biomaterials is known to improve blood compatibility [22] and enhance cell adhesion and proliferation [23]. FN, a well-characterized ECM glycoprotein, is abundant in connective tissue and plasma [24] and mediates cell-ECM interactions by binding to ECM components and integrin receptors [25]. Thin FN coatings reduce monocytes and platelet activation, making then suitable for cardiovascular implants [26]. FN coating improves biomaterial biocompatibility by promoting endothelialization via integrin-mediated binding [27]. FN is used in orthopedic and regenerative medicine applications to coat implantable materials [28]. In ophthalmology, FN-coated acrylic IOLs demonstrate improved hydrophilicity [29]. The aqueous humor contains FN at concentrations approximately 100-fold lower than plasma [30], which allows FN to remain as a stable monolayer. The FN layer naturally forming on Collamer enhances ICL biocompatibility. Anterior flare and inflammatory cell measurements obtained up to 3 yrs after Collamer insertion (293 cases, 525 eyes) showed no anterior flare or cellular reaction in >99.6% of cases [11].

Chemical surface modification of HEMA to covalently bind collagen has been shown to promote selective FN binding, mimicking the ECM microenvironment and suppressing macrophage activation while reducing non-specific protein adsorption [10]. FN’s safety and efficacy have been validated in both in vitro cell proliferation assays and in vivo transplantation studies [10].

This study has several limitations. First, we focused exclusively on FN among ECM proteins. Since FN exists in multiple isoforms, a more detailed investigation is warranted. Second, aqueous humor contains various other ECM proteins (such as laminin and type IV collagen), necessitating the analysis of all substances adhering to implanted lens surfaces. Third, the culture period was limited to 10 days, assuming it is unlikely that significant changes in adhesion or proliferation would occur beyond this timeframe, but this is unconfirmed. The use of only iris-derived cells limits generalizability, as responses of fibroblasts or immune cells were not evaluated. Furthermore, the small number of explanted cases (3 cases) from a limited patient population restricts the generalizability of findings to diverse patient populations with varying medical conditions, plus the small sample size prevented systematic analysis of the influence of implantation duration (16 vs. 171–178 months), which may be a confounding variable.

## 5. Conclusions

FN formed a membrane-like layer on the surface of Collamer, likely due to its collagen component and biological compatibility. Experimental data demonstrated greater FN adhesion to Collamer compared to IPCL, supporting the role of collagen in facilitating ECM membrane formation. Although Collamer demonstrated enhanced FN adhesion, this did not result in increased cell adhesion, presumably because HEMA-based materials have low cell-adhesive properties. However, the enhanced adhesion of FN suggests improved biocompatibility. Further studies investigating inflammatory responses, such as laser flare photometry in IPCL patients, and inclusion of larger cohort groups are needed for a more comprehensive comparison of Collamer and IPCL biocompatibility profiles.

## Figures and Tables

**Table 1:** Case information.

Case	Age/Gender	Reasons for Removal	Treatment	Lens Vault (Preoperative)	Post-Operative Uncorrected Visual Acuity
Case 1	39/Female	Due to low vault	lens replacement	52 µm	1.5 (lens vault: 1CT)
Case 2	45/Male	For monovision (OD: 0.9, OS: 0.4)	lens replacement	261 µm	OD: 1.2, OS: 1.5
Case 3	52/Male	hard to see	lens extraction	666 µm	1.2

**Table 2:** Comparison of cell activity measured by WST-8, using FN-coated LENTIS lenses as the reference.

Lens	FN Coating	Day 1	Day 5	Day 10
Collamer	Yes	32.1%	75.0%	93.8%
	No	26.8%	39.3%	70.3%
IPCL	Yes	12.5%	60.7%	66.1%
	No	12.5%	23.2%	59.4%
LENTIS	Yes	100%	100%	100%
	No	41.1%	64.3%	67.2%

## Data Availability

The dataset is available from the authors upon reasonable request.

## Supplementary Materials

The following supporting information can be downloaded at https://www.mdpi.com/article/10.3390/medicina61081384/s1, Table S1. Number of lenses used per treatment group.

## Abbreviations

The following abbreviations are used in this manuscript:

FN	Fibronectin
HEMA	Hydroxyethyl Methacrylate
ICL	Implantable Contact Lens
iHIE-NY2	immortalized human iris epithelial cells
IOL	Intraocular lens
IPCL	Implantable Phakic Contact Lens
PHAKIC-ICL	Phakic Implantable Contact Lens
WST-8	2-(2-methoxy-4-nitrophenyl)-3-(4-nitrophenyl)-5-(2,4-disulfophenyl)-2H-tetrazolium, monosodium salt
SD	Standard deviation
