# Supplementary material for: Comparison of Adhesion of Immortalized Human Iris-Derived Cells and Fibronectin on Phakic Intraocular Lenses Made of Different Polymer Base Materials
Source: Medicina (Kaunas). 2025 Jul 30;61(8):1384. doi: 10.3390/medicina61081384 (PMC12388130; doi:10.3390/medicina61081384)
Supplement: Supplementary file 1 [file medicina-61-01384-s001.zip › medicina-3686957-supplementary.pdf]

Supplementary Table S1: Number of lenses used per treatment group.

**Collamer lenses n=23**	**IPCL lenses n=22**	**LENTIS lenses n=21**	**Total lenses N=66**
**Explanted n=2** (Cases 1 & 2)	**Explanted n=1** (Case 3)	**Explanted n=0**	**Explanted N=3**
**Naïve experimental (N. exp.)** n=21<br>FN fluorescence detection (FN. fd) n=3<br>Cell adhesion experiment (C. exp) n=18	**N. exp** n=21<br>FN. fd: n=3<br>C. exp: n=18	**N. exp:** n=21<br>FN. fd: n=3<br>C. exp: n=18	**N. exp: N=63**<br>FN. fd: n=9<br>C. exp: n=54

Notes: For FNfd, each experiment used 3 lenses per type repeated twice.

For Cexp, 18 lenses per type were used (as stated in section 2.5 of the manuscript).

LENTIS served as a hydrophilic acrylic control. No explanted LENTIS lenses were analyzed.
